# Down-regulation of miR-424 inhibited the metastasis of endometrial carcinoma via targeting PTEN/PI3K/AKT signaling pathway

**DOI:** 10.7150/jca.87021

**Published:** 2023-09-04

**Authors:** Yongwei Lu, Qiaoyan Lin, Cuibo Lin, Jian Chen, Xinyan Jiang, Haixin He

**Affiliations:** 1 Department of Gynecology, Clinical Oncology School of Fujian Medical University, Fujian Cancer Hospital. Fuzhou 350014, China.; 2 Department of Blood Transfusion, Clinical Oncology School of Fujian Medical University, Fujian Cancer Hospital. Fuzhou 350014, China.

**Keywords:** Endometrial carcinoma, miR-424, PTEN, AKT, PI3K

## Abstract

**Background**: The incidence of endometrial carcinoma (EC) has been increasing annually, and treatment of advanced cases remains challenging. MicroRNA-424 (miR-424) was reported to affect several types of tumors, but its role in EC has not been studied.

**Methods**: We generated transient knockdown models of miR-424 and PTEN in EC cells. We measured mRNA and protein expression using RT-PCR and western blotting. We evaluated cell proliferation, invasion, migration, and apoptosis using CCK8, Transwell, wound healing, and flow cytometry assays. We also investigated the effect of miR-424 and PTEN on tumor growth using a metastatic tumor model in nude mice.

**Results**: The expression of miR-424 was significantly elevated in EC tissues and cell lines. MiR-424 inhibitor significantly restrained PTEN/PI3K/AKT signaling, while miR-424 mimic activated this pathway. Knockdown of PTEN significantly reversed the effects of miR-424 inhibitor on cell proliferation, invasion, migration, and apoptosis in EC cells. The significant inhibition of tumor growth and ki67 expression caused by miR-424 inhibitor were markedly promoted by sh-PTEN.

**Conclusions**: Our findings suggest that miR-424 inhibitor could inhibit cell proliferation, invasion, migration, epithelial-mesenchymal transition (EMT) process, and tumor growth, while promoting apoptosis in EC. However, the effects of miR-424 inhibitor were markedly reversed by sh-PTEN. This study provides a potential novel therapeutic strategy for the prevention and treatment of EC by targeting miR-424.

## 1. Introduction

Endometrial carcinoma (EC) accounts for 20% to 30% of malignant tumors in the female reproductive tumors 1. In recent years, the incidence rate of EC has increased year by year, and tends to be younger, ranking second in female reproductive system malignancies, and third in mortality, only second to ovarian cancer and cervical cancer 2. At present, EC is mainly treated with surgery, supplemented by radiotherapy, chemotherapy, and hormone therapy. However, the treatment of advanced patients is still difficult, with a 5-year survival rate of 16-45% and a recurrence rate of about 7.7% to 63.3% 3. Therefore, finding new targets for early diagnosis of hormone and further studying the mechanisms of its occurrence, development, and metastasis, as well as finding new treatment strategies, has become an urgent task.

MicroRNA (miRNA) is a type of single-stranded, non-coding RNA that ranges in size from 22-25 nucleotides. miRNAs play a role in regulating various cellular processes, such as cell proliferation, apoptosis, and hormone secretion, by degrading or inhibiting the translation of target mRNA through complete or incomplete complementation with miRNA 4, 5. Many studies have found that miRNA is involved in the occurrence and development of diseases, including tumors 6.

MicroRNA-424 (miR-424) is abnormally expressed in various cancers, and it is involved in affecting the biological behavior of tumors 7, 8. Previous studies have confirmed that miR-424 is associated with tumor invasion and metastasis 9. However, the mechanism of miR-424 is not yet fully understood, and there is limited research on its role in EC.

The PI3K/AKT signaling pathway is involved in biological behaviors such as apoptosis, proliferation, inflammatory response, and glucose transport during tumor development 10. Due to its clear regulatory molecules and clear mechanism research, it is widely used in various tumor mechanism studies 11. However, if miR-424 could affect the EC through targeting PI3K/AKT signaling pathway remains unclear.

In this study, we generated transient knockdown models of miR-424 and PTEN in EC cells. Our results showed that knockdown of miR-424 significantly suppressed cell proliferation, invasion, migration, epithelial-mesenchymal transition (EMT) process, while promoting cell apoptosis. The suppression of tumor size *in vivo* by miR-424 inhibitor was achieved. The cell proliferation related protein, Ki67, was suppressed in the miR424 inhibitor transfection cells. However, the effects of miR-424 were markedly reversed by sh-PTEN. Our findings suggest that targeting miR-424 may provide a novel therapeutic strategy for the prevention and treatment of EC.

## 2. Materials and methods

### 2.1 Cell culture

SHT290 (Beijing Kerisbo Biotechnology Co., Ltd, China), HEC-1-A (ATCC, US), MFE-296 (Sigma Aldrich), HEC-251 (ATCC, US) cell lines were used in this manuscript. Cells were cultivated with DMEM medium (gibco, #12491015, Langley, OK, USA) containing 5% FBS FBS (SH30071.03IR25-40, Hyclone; Cytiva, US), 40 μg/ml streptomycin, and 40 IU penicillin (V900929, Sigma, US) at 5% CO_2_ and 37℃.

### 2.2 Cell transfection

miR-424 mimic, miR-424 inhibitor, sh-PTEN, and related controls were designed and obtained from Realgene (Shanghai, China). Plasmids transfection was performed with lipofectamine 2000 (#11668019, Invitrogen, US) according to the instruction using HEC-1-A cell line. Briefly, cells were seeded into a 24-well plate (1 x 10^5^/well), and cultured under conditions of 37°C and 5% CO_2_. The cells were incubated with the transfection complexes for 48 h, and transfection efficiency was monitored. Then, cells were harvested for further analysis. Four shRNA sequences were designed to target different regions of PTEN as described previously 12, and these four shRNA sequences were concatenated in a single pLKO-PTEN-shRNA-1320 (Addgene plasmid #25638). The related sequences were listed as follows: GCAGAAACAAAAGGAGATATCA (Exon 1); GATGATGTTTGAAACTATTCCA (Exon 5); GTAGAGTTCTTCCACAAACAGA (Exon 6); GATGAAGATCAGCATTCACAAA (Exon 9).

### 2.3 Immunofluorescence staining

After reaching 90% confluence, the cells were washed with pre-heated PBS (#10010023, Gibco) 3 times for 10 minutes each time. 4% formaldehyde was used to fix cells at room temperature for 20-30 minutes. After washing with PBS 3 times (10 minutes/time), 0.2% Triton X-100 (#85111, Thermo Scientific) was used to incubate cells for 3 minutes. 5% BSA was used to incubate cells for 20 minutes. Primary antibody incubation at 4°C overnight was performed. Secondary antibody incubation at room temperature for 2h was performed. After washing with PBS 3 times (10 minutes/time), the cells were observed with a fluorescence microscope.

### 2.4 Transwell assay

The cells were seeded onto the upper chamber (#3378, Corning) with a matrix gel (#356255, Corning) with FBS-free medium. The lower chamber was added with DMEM containing 10% FBS. After 48 h, the invasive cells in the lower chamber were fixed with 70% ethanol, and stained with crystal violet (#C0775, Sigma-Aldrich) staining. Then, the invasive cells were analyzed.

### 2.5 Western blotting

The proteins were lysed using RIPA lysis buffer (meilunbio, #MA0151) containing a protein phosphatase inhibitor (meilunbio, #MB12707). First, 10% SDS-PAGE was performed, and the proteins were then transferred onto a PVDF membrane. The membrane was blocked with TBST containing 5% non-fat milk (meilunbio, #MB4219-3) for 2 hours. The membranes were subsequently incubated with primary and secondary antibodies. Then, the protein bands were analyzed. The antibodies used in this research are listed as follows: Rabbit monoclonal to PTEN (ab267787, abcam), rabbit monoclonal to PI3K (ab302958), anti-AKT (phospho T308) antibody (ab38449), anti-AKT1 + AKT2 + AKT3 antibody (ab179463), rabbit polyclonal to GAPDH (ab9485), rabbit monoclonal to N Cadherin (ab76011), rabbit monoclonal to Vimentin (ab92547), rabbit monoclonal to E Cadherin (ab40772).

### 2.6 Immunohistochemical staining

The tissues were heated in a microwave oven for 3 minutes, and incubated with 5% hydrogen peroxide for 1 minutes. The tissues were blocked with 5% non-fat milk (#MB4219-3, meilunbio), and incubated with primary antibody (rabbit polyclonal to Ki67, #ab15580). After 3 times washing with PBS (5 min/time), the second antibody (Goat anti-rabbit IgG, #ab150077) was added to incubate sections for 3 h. Then, DAB chromogenic solution was added. After dehydration and mount, the sections were captured with Olympus BX41 microscope (Tokyo, Japan). The staining intensity of IHC images was evaluated with Image J software.

### 2.7 Wound healing assay

When cells grown to 80% confluence, cells in the 6-well plate (#354721, Corning) were scraped with a 1 mL pipette. the distance between wound was tested at 0 h and 24 h, respectively. Finally, the migration distance was analyzed.

### 2.8 Flow cytometry

The digested cells were centrifuged at 2000 g for 15 min, and supernatant was discarded. Cell apoptosis kit containing propidium iodide and Annexin V-FITC (#C1062L, Beyotime, Beijing, China) were used to incubate cells in the dark for 15 min. Flow cytometry was performed to analyse cell apoptosis.

### 2.9 RT-PCR

Trizol (#R0016, Beyotime) was used to extract RNA from tissues. RNA purity was measured with Nanodrop 2000 spectrophotometer (Thermo Scientific, USA). Takara PrimeScriptrt reagent kit with gDNA eraser kit (#RR047A) was used for reverse transcription. RT-PCR was performed with Bio-Rad (CXF96). The relative expression level of gene was measured through 2^-ΔΔCT^ method. The primers are listed as follows: PTEN (F: GGGTCTGAGTCGCCTGTCA, R: CCGTGTTGGAGGCAGTAGAAG), AKT (F: ACTCATTCCAGACCCACGAC, R: AGCCCGAAGTCCGTTATCTT), PI3K (F: CTCTCCTGTGCTGGCTACTGT, R: GCTCTCGGTTGATTCCAAACT), β-actin (F: TGGCACCCAGCACAATGAA, R: CTAAGTCATAGTCCGCCTAGAAGCA, bcl-2 (F: GCTACCGTCGTCGTGACTTCGC, R: CCCCACCGAACTCAAAGAAGG), bax (F: AGACAGGGGCCTTTTTGCTAC, R: AATTCGCCGGAGACACTCG), caspase-3 (F: CTCGCTCTGGTACGGATGTG, R: TCCCATAAATGACCCCTTCATCA), N-cadherin (F: CACCATGTGCCGGAAAGAGCCCTT, R: AGTTCAGTCGTCGCTCCCTCCGTACAT, E-cadherin (F: TACACTGCCCAGGAGCCAGA, R: TGGCACCAGTGTCCGGATTA, vimentin (F: GACGCCATCAACACCGAGTT, R: CTTTGTCGTTGGTTAGCTGGT. The adjacent normal tissues and tumor tissues from 20 endometrial carcinoma patients in our hospital were collected. The mRNA levels of miR-424 in tissues were measured with RT-PCR.

### 2.10 CCK8 assay

The cells were plated into 96-well, and their cellular ability was evaluated after 12 hours. Following a 30-minute incubation with CCK8 reagent (Beyotime, #C0038, China), the cell proliferation ability was measured.

### 2.11 Dual-luciferase reporter assay

TargetScan data were utilized to predict the binding site between PTEN and miR-424. Subsequently, a luciferase reporter assay was performed. Wild-type and mutant reporters (PTEN-WT and PTEN-MUT), along with NC mimics and miR-424 mimics, were co-transfected into cells using Lipofectamine 2000. Dual-luciferase reporter assay was performed according to the instruction. PTEN-sh-mut (5'-CGCGGATCCAAATATTACATTA-AGGGTTAAGTGATTTGATTCAGTAATTGTGCTATTCCTA-CATGTGCTTTATAGATTAAAGAATTGATTTTTTGG-TACCCCG-3') were synthesized by Augct (Beijing, China). The PTEN 3'UTR fragments were inserted downstream of the control vector (Promega, USA) via Fse I and Xba I sites.

### 2.12 Statistical analysis

The data were presented with mean ± standard deviation. Data were statistically analyzed using SPSS (Version 18). p-value < 0.05 was set as statistical difference. T-test and ANOVA test was used for statistical analysis.

## 3. Results

### 3.1 The expression of miR-424 was significantly elevated in the EC tissues and cell lines

**Figure 1:**
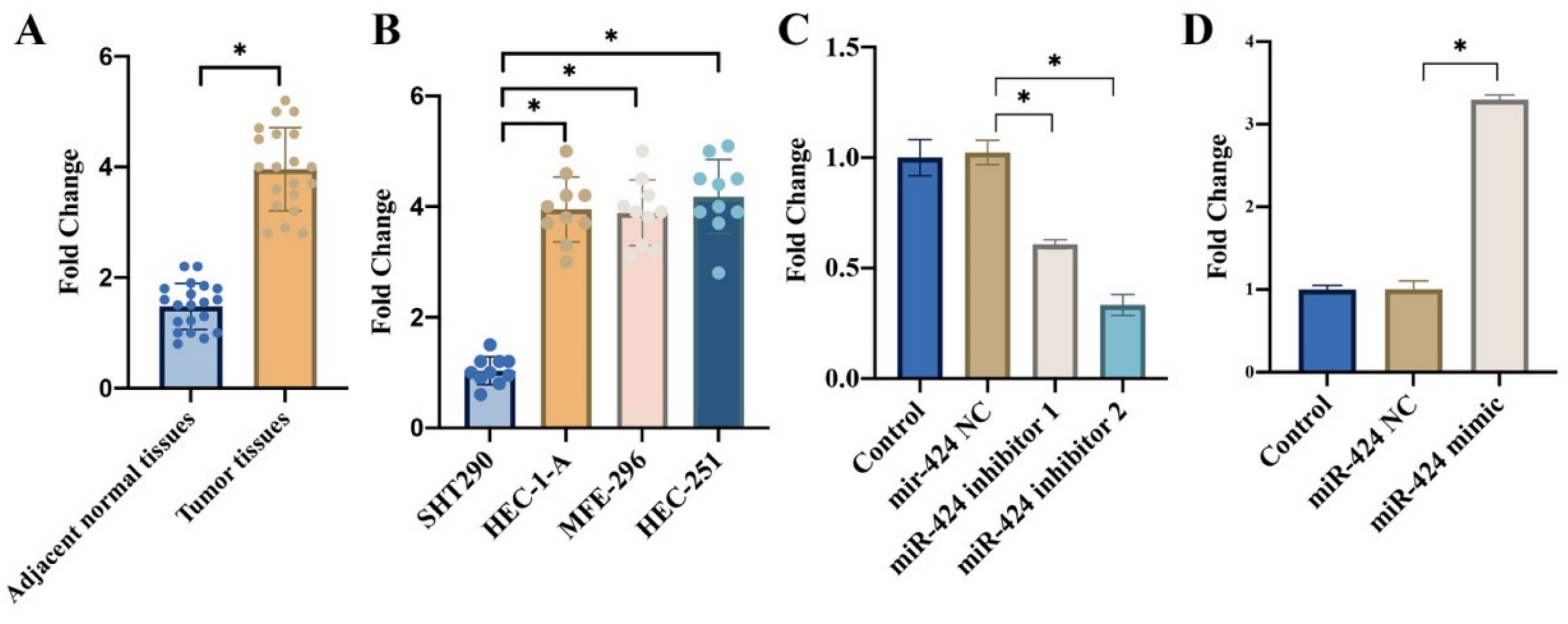
**The expression of miR-424 was significantly elevated in the EC tissues and cell lines*.*** (A) Remarkable higher mRNA expression of miR-424 was observed in the EC tissues; (B) Remarkable higher mRNA expression of miR-424 was observed in EC cell lines; (C) The transient knockdown of miR-424 in cells were constructed in HEC-1-A cell line, and the mRNA level of miR-424 was evaluated (n=3); (D) The transient overexpression of miR-424 in cells were constructed in HEC-1-A cell line, and the mRNA level of miR-424 was evaluated (n=3). * means p <0.05.

We found that remarkable higher expression of miR-424 was observed in the EC tissues (Figure 1 A) and cell lines including HEC-1-A, MFE-296, HEC-251 (Figure 1 B) compared with related controls. In addition, the transient knockdown and overexpression of miR-424 in cells were constructed with HEC-1-A cell line (Figure 1 C-D).

### 3.2 miR-424 inhibitor greatly suppressed PTEN/PI3K/AKT, but miR-424 mimic markedly activated this signaling pathway

**Figure 2:**
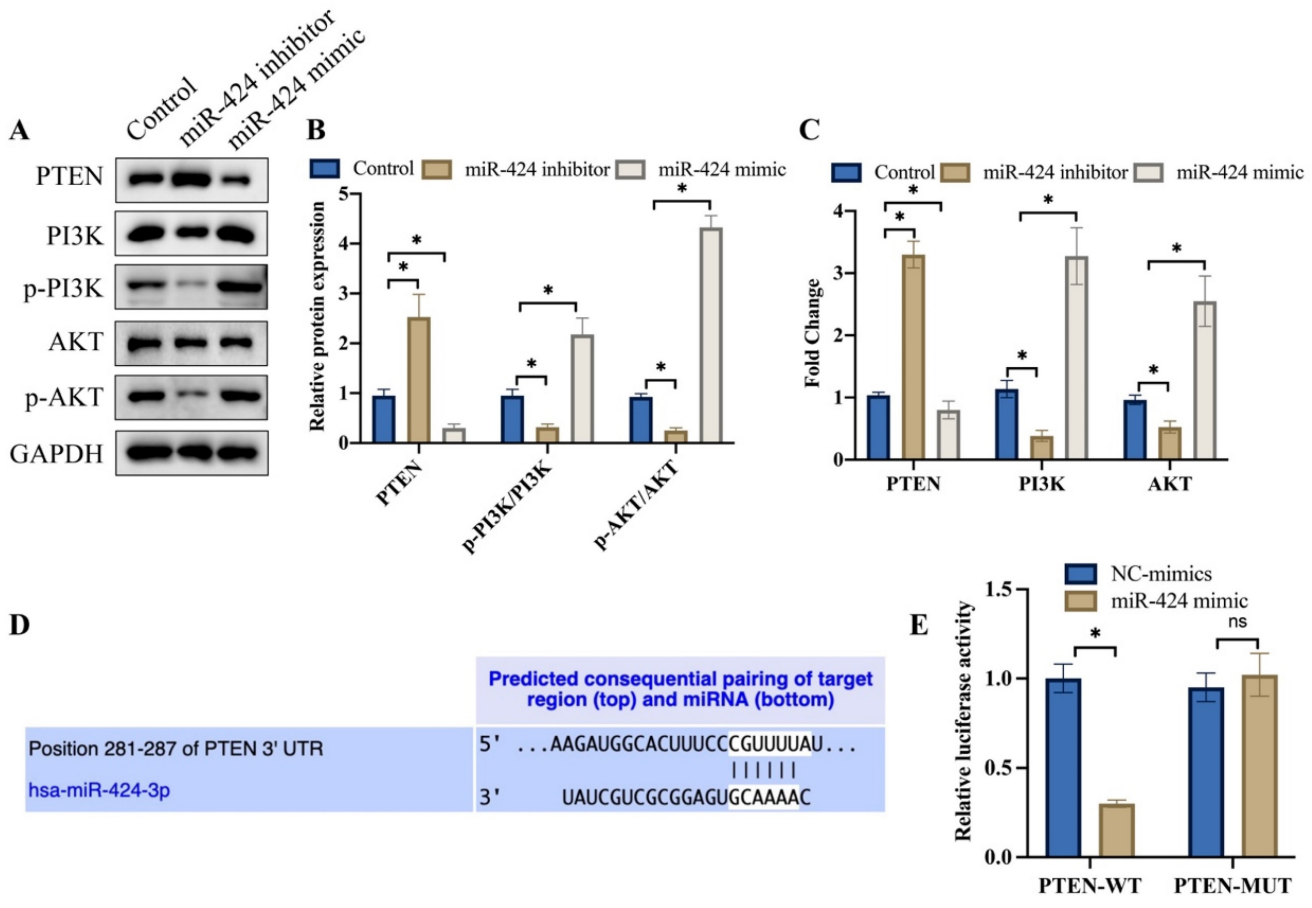
**miR-424 inhibitor greatly suppressed PTEN/PI3K/AKT, but miR-424 mimic markedly activated this signaling pathway*.*** (A) The protein expression of PTEN/PI3K/AKT was detected with western blotting; (B) The protein expression was analyzed; (C) The mRNA levels of PTEN/PI3K/AKT were measured with RT-PCR; (D) The binding site between miR-424 and PTEN was predicted with TargetScan data base; (E) The luciferase activity of PTEN-WT was inhibited by miR-424. * means p <0.05. The experiment was performed 3 times.

PTEN/PI3K/AKT is closely linked with the progression of tumor, and we want to investigate the influence of miR-424 on this signaling pathway. We found that knockdown of miR-424 greatly suppressed PTEN/PI3K/AKT (Figure 2 A-C), but overexpression of miR-424 significantly activated PTEN/PI3K/AKT signaling pathway (Figure 2 A-C). The binding site between miR-424 and PTEN was predicted with TargetScan data base (Figure 2 D). The luciferase activity of PTEN-WT was inhibited by miR-424 (Figure 2 E).

### 3.3 Knockdown of PTEN significantly reversed the effects of miR-424 inhibitor on cell proliferation, invasion, and migration of EC cells

**Figure 3:**
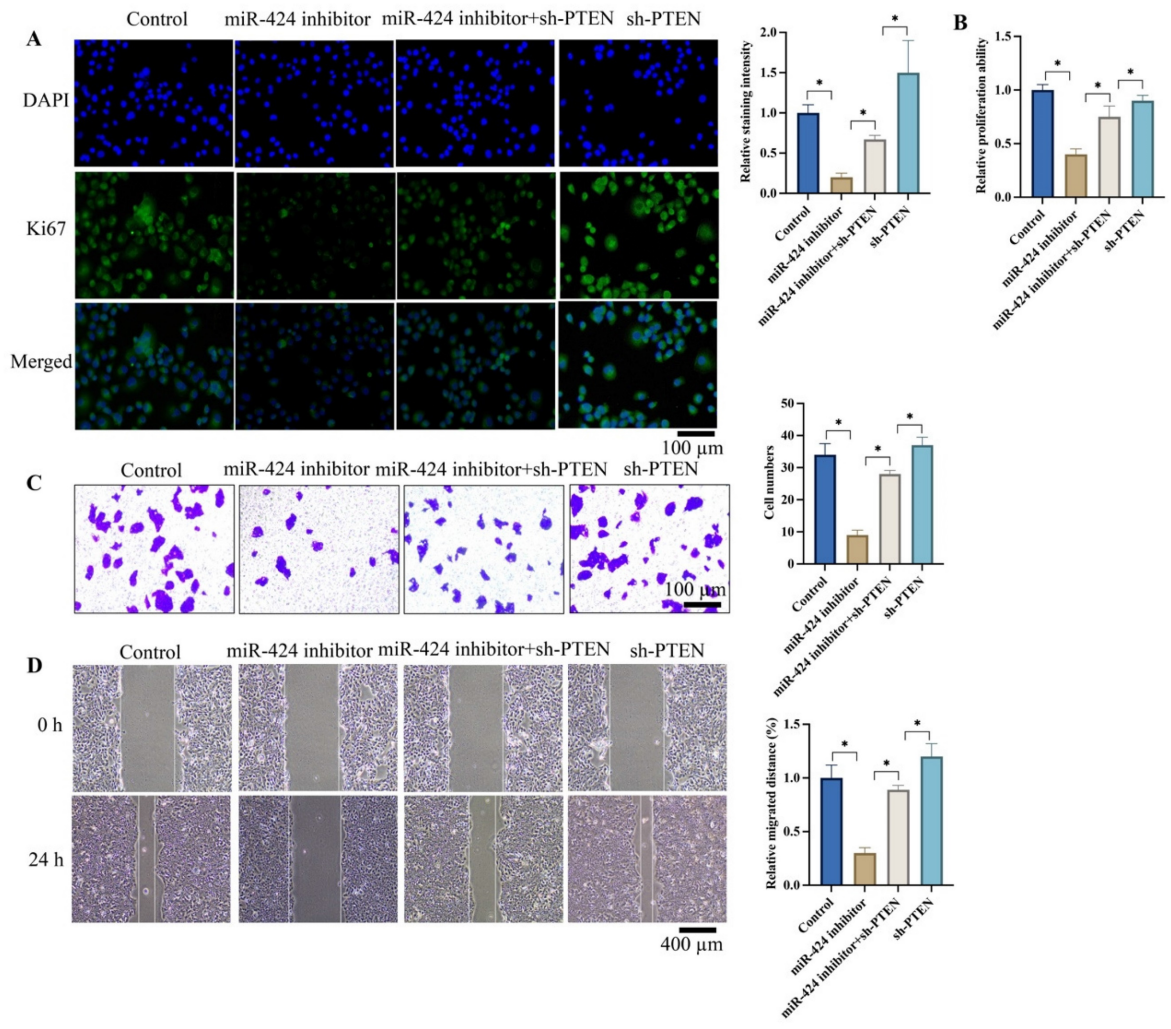
** Knockdown of PTEN significantly reversed the influence of miR-424 inhibitor on cell proliferation, invasion, and migration of EC cells.** (A) The Ki67 expression in cells were evaluated; (B) The cell proliferation ability was detected with CCK8 assay; (C) The cell invasion ability was evaluated with Transwell assay; (D) The cell migration ability was evaluated with wound healing assay. * means p <0.05. The experiment was performed 3 times.

Ki67 expression intensity represents cell proliferation ability. We found that miR-424 inhibitor markedly suppressed Ki67 expression (Figure 3A) and cell proliferation (Figure 3B) evaluated by CCK8 assay. However, sh-PTEN greatly promoted Ki67 expression (Figure 3A) and cell proliferation (Figure 3B) compared to the miR-424 inhibitor group. Additionally, sh-PTEN significantly increased the decreased cell invasion (Figure 3C) and migration (Figure 3D) caused by miR-424 inhibitor (Figure 3C-D).

### 3.4 Knockdown of PTEN significantly reversed the effects of miR-424 inhibitor on cell apoptosis, cell cycle stage, and EMT process

**Figure 4:**
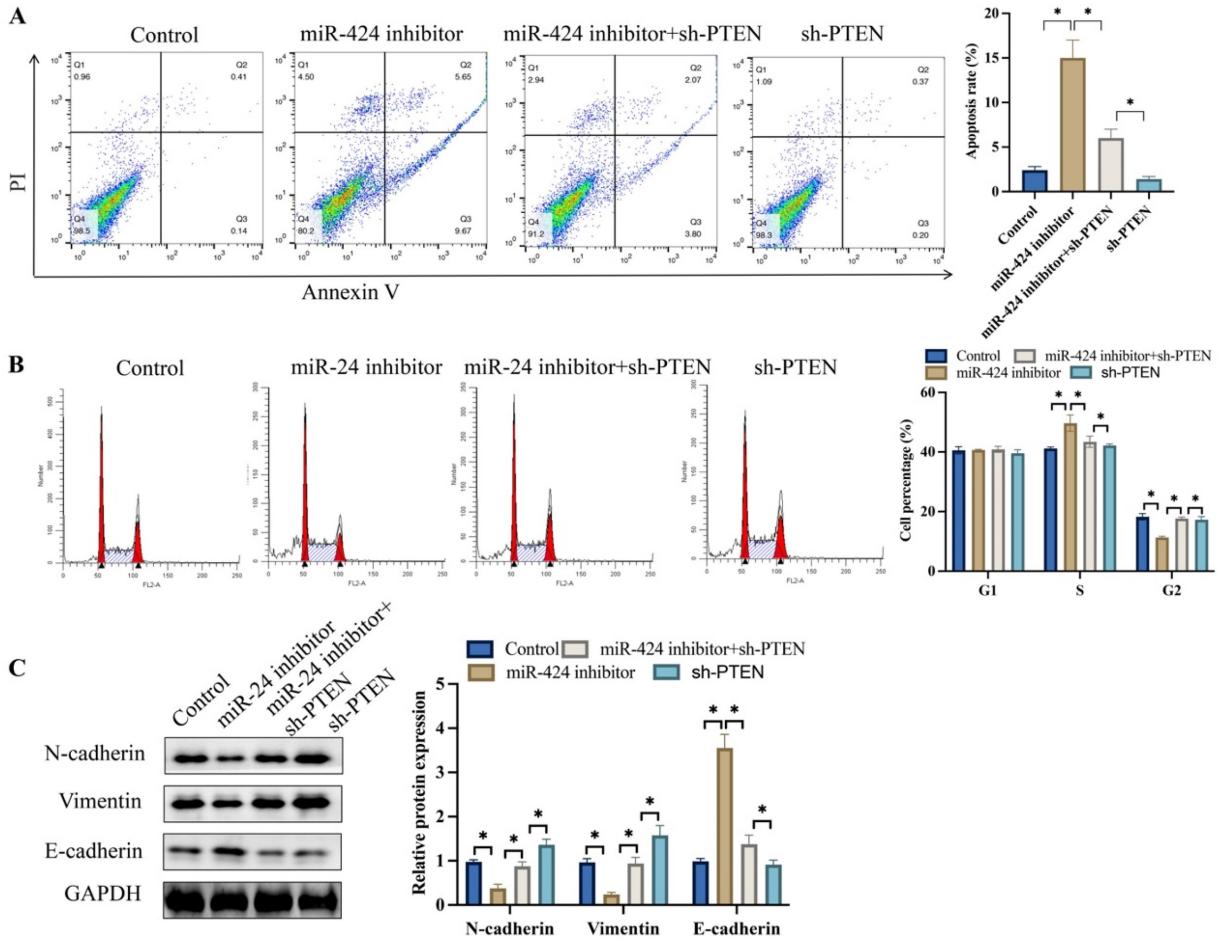
** Knockdown of PTEN significantly reversed the influence of miR-424 inhibitor on cell apoptosis, cell stage, and EMT process.** (A) Cell apoptosis was measured with flow cytometry; (B) The cell cycle change was measured with flow cytometry; (C) The protein levels of EMT proteins were detected with western blotting. * means p <0.05. The experiment was performed 3 times.

Meanwhile, tumor cell apoptosis was detected after transfection with miR-424 inhibitor. Knockdown of PTEN markedly inhibited cell apoptosis compared to the miR-424 inhibitor group (Figure 4A). In addition, miR-424 inhibitor markedly blocked cells in the S stage, and the percentage of cells in the G2 stage was greatly decreased (Figure 4B). However, simultaneous transfection with sh-PTEN significantly reversed the effects of miR-424 inhibitor and increased the percentage of cells in the G2 stage (Figure 4B). The EMT process was inhibited after transfection with miR-424 inhibitor but was promoted after simultaneous treatment with sh-PTEN (Figure 4C).

### 3.5 Knockdown of PTEN significantly reversed the effects of miR-424 inhibitor on tumor growth in vivo

**Figure 5:**
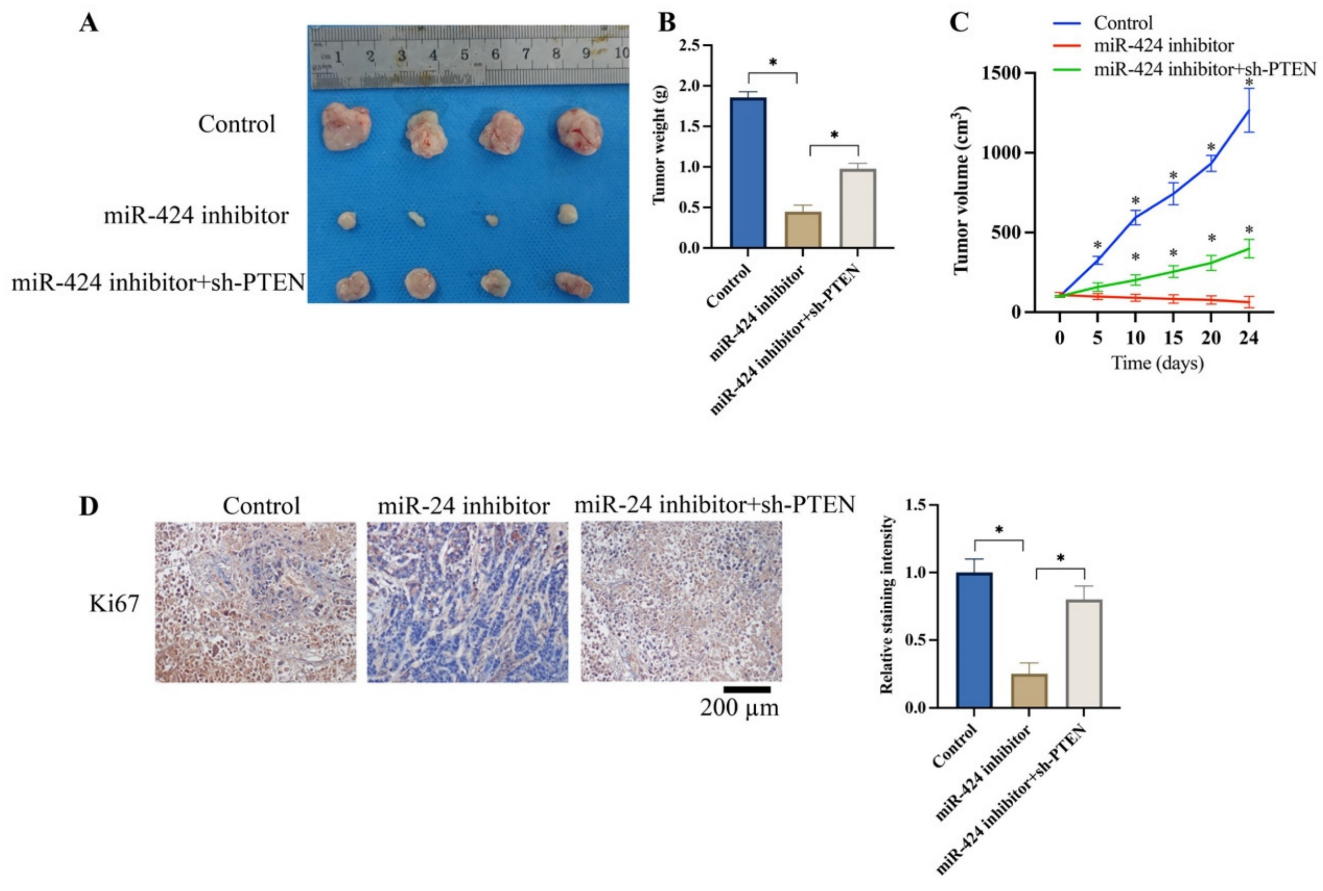
** Knockdown of PTEN significantly reversed the influence of miR-424 inhibitor on tumor growth *in vivo*.** (A) Metastatic tumor experiment was performed (n=4); (B) The tumor weight was recorded; (C) The tumor volume was analyzed (* means p<0.05 compared with group miR-424 inhibitor); (D) The ki67 expression in the tumor tissue was evaluated (n=4). * means p <0.05.

We investigated the effects of miR-424 and PTEN on tumor growth in vivo. The tumor weight (Figure 5A-B) and volume (Figure 5C) were significantly reduced after knockdown of miR-424 inhibitor. Meanwhile, the expression of Ki67 in the tumor tissue was suppressed by miR-424 inhibitor (Figure 5D). However, the remarkable inhibition of tumor growth and Ki67 expression were increased by sh-PTEN (Figure 5A-D).

### 3.6 Knockdown of PTEN significantly reversed the effects of miR-424 inhibitor on the EMT process in vivo

**Figure 6:**
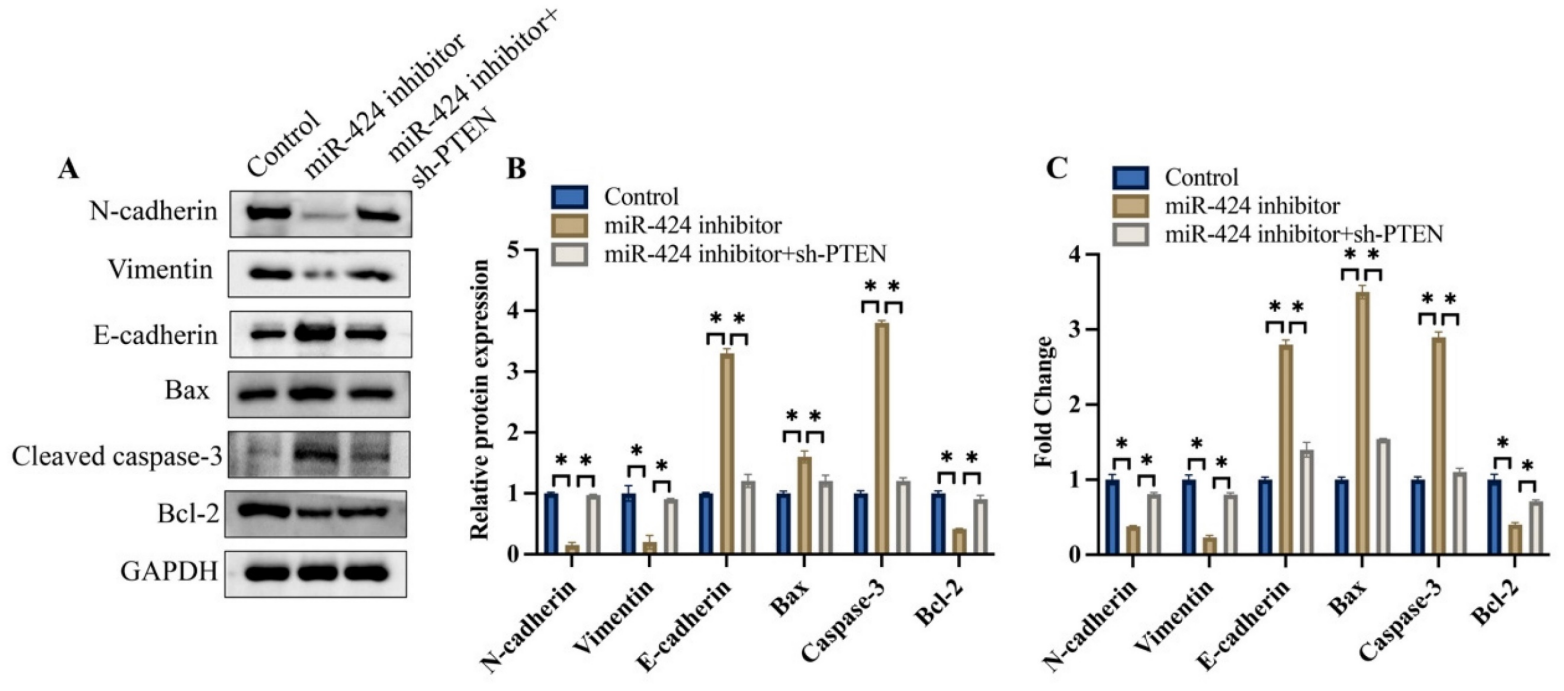
** Knockdown of PTEN significantly reversed the influence of miR-424 inhibitor on EMT process *in vivo*.** (A) The EMT and apoptosis related proteins were measured with western blotting; (B) The protein expression was analyzed; (C) The mRNA expression of EMT and apoptosis related proteins was measured with RT-PCR. * means p <0.05. The experiment was performed 3 times.

We also investigated the effects of miR-424 and PTEN on the EMT process and apoptosis-related proteins in vivo. We found that the expression of N-cadherin and vimentin was inhibited, but E-cadherin was increased after transfection with miR-424 inhibitor, but was promoted after simultaneous treatment with sh-PTEN (Figure 6A-C). Meanwhile, pro-apoptotic proteins, including bax and caspase-3, were increased by miR-424 inhibitor, but the anti-apoptotic protein bcl-2 was decreased. However, the effects of miR-424 inhibitor on EMT and apoptosis-related proteins were reversed by sh-PTEN (Figure 6A-C).

## 4. Discussion

miRNA is an endogenous, non-coding, small molecule RNA that regulates gene expression by complementary pairing with mRNA bases. This process affects various biological processes, such as cell proliferation, invasion, and angiogenesis 13. Bioinformatics predicts that approximately 1%-5% of protein-coding genes in organisms are regulated by miRNAs, and one-third of human protein-coding genes are regulated by miRNAs 14. Each miRNA has hundreds of target genes, and the same target gene can be regulated by multiple miRNAs 15. This indicates that miRNA is one of the largest regulatory gene families. Currently, the regulatory role of miRNAs in tumors has been widely reported 16.

miR-424 belongs to the miR-16 family and is located on chromosome Xq26.3. The Xq26 region plays an important role in cell cycle, proliferation, apoptosis, and survival, and is associated with the occurrence and development of various tumors 17. In recent years, studies have found that miR-424 is abnormally expressed in a variety of cancers, but it is highly expressed in pancreatic cancer and renal clear cell carcinoma 18. Studies indicated that overexpression of miR-424 in liver cancer cells directly maintained E-cadherin/ β-Catenin complex on the cell membrane, and prevented epithelial mesenchymal transformation 19. Tumor inhibitor miR-424 abrogates ferroptosis in ovarian cancer through targeting ACSL4 4. However, miR-424 coordinates multilayered regulation of cell cycle progression to promote esophageal squamous cell carcinoma 20. Therefore, the regulatory role of miR-424 in tumor is complicated. We demonstrated that miR-424 was tumor promotor in EC. Further study about the deep role of miR-424 in tumors need to be explored.

Currently, there are few reports on the biological mechanisms of miR-424. The mechanism by which miR-424 participates in tumor invasion and metastasis mainly focuses on related target molecules and their downstream MAPK, ERK, and HIF signal transduction pathways, which regulate cell cycle and apoptosis, ultimately affecting cell growth, invasion, and metastasis 21-23. miR-424 has multiple targets and extensive functions, and its mechanism requires further exploration 24, 25. In this study, we demonstrated that knockdown of PTEN could reverse the effects of miR-424 on the malignancy of EC, indicating that miR-424 might regulate EC through targeting the PI3K/AKT signaling pathway.

The PI3K/AKT signaling pathway has been shown to play an important role in the initiation, progression, and deterioration of tumors. The bromodomain PHD finger transcription factor in bone marrow provides a potential progression biomarker regulated by TFAP4 through the PI3K/AKT pathway in neuroblastoma 26. Activation of the PI3K/AKT pathway is a potential mechanism of treatment resistance in small cell lung cancer 27. In this research, knockdown of miR-424 significantly suppressed the PI3K/AKT signaling pathway, and transfection with sh-PTEN markedly reversed the effects of miR-424 inhibitor on EC metastasis and growth.

## 5. Conclusion

In summary, we proved that miR-424 inhibitor could suppress the cell proliferation, invasion, migration, EMT process, tumor growth, but promoted cell apoptosis. However, the influence of miR-424 inhibitor was markedly reversed by sh-PTEN. This study might provide a novel therapeutic strategy for the prevention and treatment of EC through targeting miR-424.

## Abbreviations

EC): Endometrial carcinoma
miRNA): microRNA
miR-424): microRNA-424
EMT): epithelial mesenchymal transition
